# Foundation model-based haemoglobin concentration estimation and anaemia risk assessment from retinal fundus images

**DOI:** 10.1038/s41433-026-04743-3

**Published:** 2026-07-22

**Authors:** Sangmin Kwak, Jaewon Kim, Hyeokjong Lee, Changho Han, Jooyoung Chang, Sang Min Park

**Affiliations:** 1 College of Medicine, Seoul National University, Seoul, Republic of Korea; 2 Department of Biomedical Sciences, Seoul National University Graduate School, Seoul, Republic of Korea; 3 Medical Big Data Research Center, Seoul National University Medical Research Center, Seoul National University College of Medicine, Seoul, Republic of Korea; 4 XAIMED Co. Ltd, Seoul, Republic of Korea

**Keywords:** Predictive markers, Predictive markers

## Abstract

**Introduction:**

Widespread screening of anaemia is currently limited by the need for invasive blood tests. This study’s primary objective was to evaluate the feasibility and interpretability of Vision Transformer (ViT)-based foundation models, including those pretrained on natural or medical domains, for the noninvasive estimation of haemoglobin (Hb) levels using retinal fundus images.

**Methods:**

This retrospective cross-sectional study included 34,511 individuals from the Health Promotion Center of Seoul National University Hospital (2005–2016). We adapted five foundation models (DINOv2, OpenCLIP, MAE, RETFound, and VisionFM) for Hb concentration regression using low-rank adaptation (LoRA) and partial fine-tuning. For explainability, we applied Grad-CAM saliency mapping and perturbation-based analysis using retinal vessel segmentation.

**Results:**

Among all tested models, the natural-domain model DINOv2 combined with LoRA achieved the best performance (MAE = 0.703, R^2^ = 0.682, and AUROC = 0.917 for anaemia detection). Operating characteristics were examined at fixed specificity levels across different haemoglobin thresholds. Grad-CAM analysis revealed that the model’s predictions predominantly relied on the macula and peripapillary regions. Perturbation analysis confirmed that increased brightness and colour saturation of retinal arteries correlated with higher estimated Hb levels.

**Conclusions:**

Fine-tuned foundation models, particularly DINOv2 with LoRA, demonstrated competitive performance in noninvasive haemoglobin estimation from retinal fundus images. The model’s decision-making process was found to be qualitatively consistent with clinical and physiological expectations, with predictions focusing on known anatomical and vascular indicators. These preliminary findings suggest the potential use of adapted foundation models as adjunctive tools for anaemia risk assessment, though external validation remains necessary.

## Introduction

Haemoglobin, the concentration of which is usually measured using blood tests, is an important protein in red blood cells that carries oxygen [1]. Abnormalities in haemoglobin concentration are well known to indicate various underlying conditions [2] and to be associated with an increased risk of cardiovascular events and all-cause mortality [3, 4]. Anaemia, characterised by insufficient haemoglobin levels, affected 24.3% of the global population, equivalent to 1.92 billion people, in 2021 [5]. From a clinical perspective, anaemia can be a strong indicator of colorectal cancer; therefore, guidelines in the EU, UK, and US recommend follow-up monitoring of patients with anaemia [6–8]. However, the diagnosis of anaemia relies on invasive blood testing, which presents several limitations, including patient discomfort, procedural complications such as infection or vascular injury, and resource constraints in healthcare delivery [9].

The emergence of noninvasive haemoglobin measurement techniques represents a promising advancement in addressing these limitations by enabling real-time monitoring, reducing patient discomfort, and enhancing accessibility in resource-limited settings [10–12]. In particular, deep learning has demonstrated considerable potential for noninvasive haemoglobin assessment using various types of images, such as retinal fundus, ultrawide-field fundus, and conjunctival images [13–17].

Developing a deep learning model from scratch requires substantial computational resources, large datasets, and expert knowledge of model architecture. Fine-tuning a pretrained general-purpose model significantly reduces these demands. Foundation models (FMs) produce universal features applicable across diverse computer vision tasks [18]. FMs have also shown great potential in medical artificial intelligence, offering capabilities for processing and analysing medical imaging data [19–22].

However, the efficacy and specific challenges of fine-tuning FMs in a medical settings have not yet been comprehensively analysed. Specifically, the potential of FMs pretrained on natural images for estimating physiological parameters, such as haemoglobin concentrations from retinal fundus photographs, remains largely unexplored. To address this critical gap, we evaluated the performance of various FMs after fine-tuning them on retinal fundus images to estimate haemoglobin concentrations in a cohort of adult Korean adults. Furthermore, to ensure clinical relevance, we sought to explain the model’s decision-making process using saliency maps [23] and perturbation methods.

## Methods

### Data collection

**Fig. 1:**
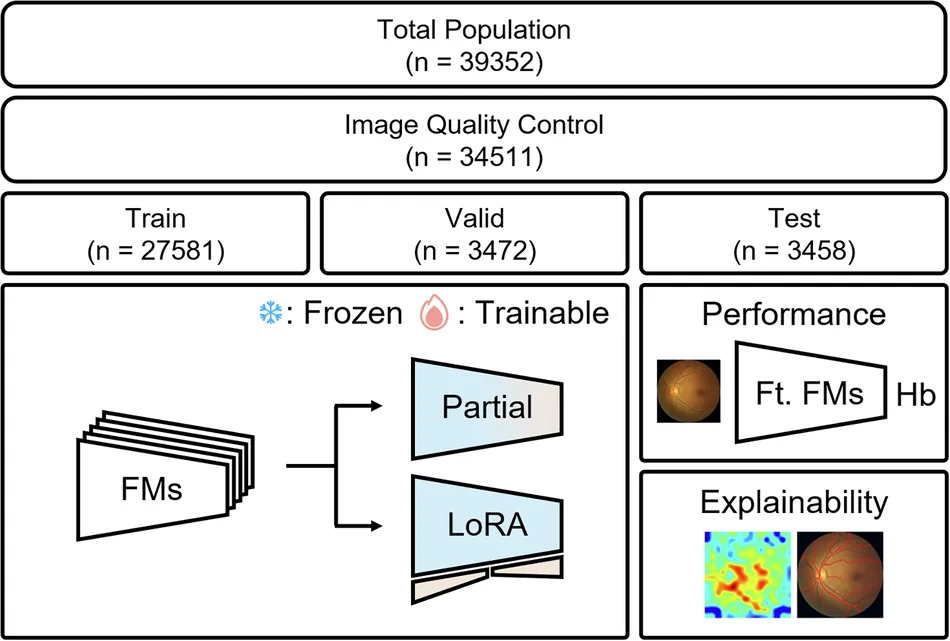
The overall flow chart of study design. FMs Foundation models, Partial Partial finetuning, LoRA LoRA finetuning, Ft. Fine-tuned, Hb haemoglobin.

Figure 1 describes the overall schema. The study data from participants who had undergone medical examinations from the Health Promotion Center of Seoul National University Hospital (HPC-SNUH) between January 2005 and December 2016, as reported in a previous study [24]. The HPC-SNUH offers several medical examination protocols consisting of a survey, physical examinations, laboratory testing, and medical imaging. Retinal fundoscopy and complete blood count (CBC) were included in all packages. Retinal fundoscopy was performed via a CR-2 digital non-mydriatic fundus camera (Canon, Tokyo, Japan) [25]. The procedure was conducted without pupil dilation, and a single colour fundus image was captured for each eye. All images were acquired using a fixed 45-degree field of view covering both the optic disc and macula to standardise the image acquisition protocol. The individuals were randomly split at an 8:1:1 ratio for training, validation, and testing, maintaining subject-level separation to prevent data bias. For patients with multiple visits, only the most recent visit was included (*n* = 39,352). To maintain acceptable data quality, we implemented an automated quality assessment using AutoMorph [26], a deep learning model pretrained on the EyePACS dataset. The model was trained to assess images based on three criteria, namely, illumination quality, the presence of artifacts, and overall diagnosability for common eye diseases, as validated by expert ophthalmologists. Approximately 19.3% of the retinal fundus images were excluded after quality control (*n* = 34,511). Following WHO guidelines, we set all cutoff values on the basis of haemoglobin (Hb) measurements. Anaemia was defined using sex-specific thresholds (<12 g/dL in women and <13 g/dL in men). Among anaemic individuals, mild anaemia was defined as Hb 11.0–12.0 g/dL in women and 11.0–13.0 g/dL in men, moderate anaemia as 8.0–11.0 g/dL, and severe anaemia as <8.0 g/dL. Clinically significant anaemia was defined as moderate-to-severe anaemia (Hb <11 g/dL), representing a clinically relevant subset of all anaemia cases. To confirm the consistency of the results, we evaluated each model’s performance in subgroups defined by sex, age, and comorbidities with hypertension (*n* = 2110), diabetes mellitus (*n* = 1087), and dyslipidaemia (*n* = 3011) within the test dataset. Each subgroup was defined based on a combination of clinical data, including laboratory measurements (e.g., fasting blood glucose, HbA1c, systolic and diastolic blood pressure, total cholesterol, LDL cholesterol, HDL cholesterol, and triglycerides), current medication use, and prior diagnostic history.

### Foundation models

We employed the following FMs based on a Vision Transformer (ViT) architecture [27], as detailed in Supplementary Table 1:

DINOv2 [28] was trained on a curated dataset of 142 M natural images from the web. The model excels in general visual representation learning across diverse computer vision tasks. OpenCLIP [29] is built upon CLIP’s vision-language architecture and uses a ViT for images and a transformer for text. We used a version trained on LAION-2B, an English subset of LAION-5B [30] consisting of image-text pairs from the web. MAE [31] uses an asymmetric encoder-decoder vision transformer architecture pretrained on the ImageNet-1K dataset, with the encoder operating only on visible patches and a lightweight decoder reconstructing masked patches. RETFound [32] is a ViT architecture specifically pretrained on a large-scale dataset of 1.5 M retinal images from diverse sources, including fundus photographs and OCT scans. We used a version trained with 904 K retinal fundus images. VisionFM [33] was trained on a comprehensive dataset of 3.4 M ophthalmic images across multiple modalities for disease screening, diagnosis, prognosis prediction, subclassification, and even systemic biomarker and disease prediction.

### Fine-tuning methods

We used partial fine-tuning and low-rank adaptation (LoRA) [34] to fine-tune FMs for estimating haemoglobin levels from retinal fundus images. Partial fine-tuning [35] enhances a model’s adaptability by training only the last few transformer blocks and the classification head whilst keeping the earlier encoder layers frozen. This approach builds on the concept of feature reuse, where initial layers extract general patterns such as edges and textures, and deeper layers learn task-specific representations. In this study, we restricted training to the final block only. LoRA is a memory-efficient fine-tuning method that introduces small, trainable low-rank matrices into the pretrained transformer layers. Instead of updating the entire model, LoRA adjusts only a limited set of additional parameters, preserving the original model’s architecture. This makes it particularly effective for adapting large pretrained models to specific domains without requiring full retraining.

### Implementation details

All model development and training procedures were implemented using PyTorch [36] in Python 3.10 on four NVIDIA 3090 GPUs. For all foundation models, the original classification head was replaced with a single-output fully connected layer for haemoglobin regression, whilst the backbone architectures were kept unchanged. As part of preprocessing, all fundus images were resized to 224 × 224 and then centre-cropped prior to model input, ensuring consistent spatial alignment of anatomical regions across images. We implemented comprehensive data augmentation strategies during training, including RandAugment and random erasing, following the protocols established in the RETFound framework [32]. We used a batch size of 32 for partial fine-tuning and 8 for LoRA tuning, training each model for 100 epochs. The learning rate followed a warm-up schedule and increased to 5e−4 during the first 10 epochs, followed by cosine annealing. We employed the AdamW optimiser with a weight decay of 1e−3 and used mean squared error as the loss function for haemoglobin prediction.

### Evaluation of predictive performance

We compared the performance of various models and fine-tuning methods for predicting haemoglobin concentration and for characterising their operating characteristics with respect to anaemia-related outcomes. Based on the predicted haemoglobin values, anaemia was defined using sex-specific thresholds (<12 g/dL for women and <13 g/dL for men). Clinically significant anaemia was defined using a lower haemoglobin threshold (<11 g/dL). Proportion-based metrics, including sensitivity, specificity, and accuracy, were calculated from the confusion matrix, and their 95% confidence intervals were estimated using the Wilson score method. For MAE, R², and AUROC, 95% confidence intervals were estimated using 1000 bootstrap resamples with replacement, and percentile-based two-sided intervals were reported. To further characterise the operating characteristics of the best-performing model, we evaluated sensitivity at predefined specificity levels for any anaemia and reported the corresponding predicted haemoglobin thresholds. In addition, positive predictive value (PPV) was calculated across percentiles of predicted haemoglobin values for the best-performing model.

### Explaining the clinical rationale of the model

To explain the model’s clinical rationale, we employed two methods: the saliency-map method and the perturbation-based approach. For the saliency-map method, Grad-CAM [37] was utilised to visualise the regions of interest in retinal images that influenced the model’s predictions. To generalise the results, we averaged all saliency maps across the test dataset, separately for the left and right eye. To further investigate the model’s response to vessel characteristics, we employed segmentation data obtained from the AutoMorph [26] module to isolate arteries. We then modified the brightness and saturation of the images either in artery-only regions or across the entire images. For each condition, we recorded the mean predicted haemoglobin values and their corresponding standard deviations to assess how changes in arterial appearance influenced the model’s predictions.

### Ethics

This dataset was obtained with the approval of the Institutional Review Board of Seoul National University Hospital (IRB number: H-1703-044-837), which waived the requirement for patient consent. All patient data were anonymised prior to analysis.

## Results

### Study population and characteristics

The study included a total of 63,528 images from 34,511 Korean individuals, distributed across training (50,758 images, 27,581 subjects), validation (6419 images, 3472 subjects), and test sets (6351 images, 3458 subjects). As summarised in Table 1, the demographic characteristics were well balanced across all sets, with a mean age of ~58 years (training: 57.84 ± 10.48, validation: 58.13 ± 10.44, test: 57.76 ± 10.62) and a balanced gender distribution (~49% female). The mean haemoglobin concentrations were also similar across sets (training: 14.46 ± 1.58 g/dL, validation: 14.50 ± 1.59 g/dL, test: 14.45 ± 1.59 g/dL). Anaemia prevalence in the detailed subgroups is presented in Supplementary Table 2.

**Table 1:** Baseline characteristics of the dataset.

	Train	Valid	Test
Images	50,758	6419	6351
Subject population	27,581	3472	3458
Age(years), mean ± SD	57.84 ± 10.48	58.13 ± 10.44	57.76 ± 10.62
Hb(g/dL), mean ± SD	14.46 ± 1.58	14.5 ± 1.59	14.45 ± 1.59
Females(%)	49.06	48.43	49.08
Population, *n* (%)			
No anaemia	26,199 (94.99)	3300 (95.05)	3285 (95.00)
Mild anaemia	971 (3.52)	111 (3.20)	122 (3.53)
Moderate anaemia	377 (1.37)	55 (1.58)	47 (1.36)
Severe anaemia	34 (0.12)	6 (0.17)	4 (0.12)

Baseline characteristics of individuals in the training, validation, and test datasets. Values are presented as mean ± standards deviation for continuous variable and as number (percentage) for categorical variables.

### Performance of the models for predicting haemoglobin levels

As shown in Table 2, in terms of haemoglobin estimation performance (MAE, R-squared), the LoRA tuning method generally proved superior to partial finetuning. The DINOv2 model with LoRA tuning achieved the best overall estimation results, recording the lowest MAE (0.703) and the highest R-squared (0.682). However, the RETFound model was an exception, with its partial finetuning (MAE 0.787, R-squared 0.606) performed marginally better than its LoRA tuning counterpart. For anaemia-related outcomes, DINOv2 with LoRA tuning achieved the highest AUROCs for anaemia (0.917) and clinically significant anaemia (0.982), indicating superior discrimination based on predicted haemoglobin values. However, the number of individuals with clinically significant anaemia in the test set was limited (*n* = 51), and these results should be interpreted with caution. Supplementary Fig. 1 presents Bland–Altman and linear regression analyses for this best-performing model, showing a small mean difference (−0.08 g/dL) and a positive association between predicted and measured haemoglobin concentrations.

**Table 2:** Predictive performance of models for haemoglobin estimation and anaemia prediction.

Model	Finetuning method	Estimation of haemoglobin		Prediction of anaemia				
		MAE (95% CI)	R squared (95% CI)	Accuracy (95% CI)	Sens. (95% CI)	Spec. (95% CI)	AUROC (95% CI)	AUROC (cSA) (95% CI)
DINOv2	Partial Finetuning	0.892 (0.876–0.911)	0.482 (0.464–0.500)	0.950 (0.944–0.955)	0.089 (0.062–0.126)	0.994 (0.992–0.996)	0.814 (0.791–0.835)	0.912 (0.882–0.936)
	LoRA tuning	0.703 (0.688–0.717)	0.682 (0.667–0.696)	0.957 (0.952–0.962)	0.363 (0.312–0.418)	0.988 (0.985–0.991)	0.917 (0.903–0.929)	0.982 (0.975–0.989)
OpenCLIP	Partial Finetuning	0.908 (0.890–0.926)	0.470 (0.449–0.488)	0.951 (0.945–0.956)	0.118 (0.087–0.158)	0.994 (0.992–0.996)	0.804 (0.779–0.827)	0.920 (0.892–0.944)
	LoRA tuning	0.752 (0.738–0.767)	0.638 (0.622–0.652)	0.954 (0.949–0.959)	0.274 (0.228–0.326)	0.989 (0.986–0.992)	0.890 (0.873–0.906)	0.959 (0.940–0.976)
MAE	Partial Finetuning	0.795 (0.779–0.810)	0.591 (0.571–0.607)	0.954 (0.948–0.959)	0.229 (0.186–0.279)	0.992 (0.989–0.994)	0.874 (0.855–0.893)	0.957 (0.937–0.974)
	LoRA tuning	0.739 (0.725–0.754)	0.650 (0.634–0.664)	0.955 (0.950–0.960)	0.338 (0.288–0.392)	0.987 (0.984–0.990)	0.904 (0.888–0.918)	0.977 (0.967–0.986)
RetFound	Partial Finetuning	0.787 (0.773–0.803)	0.606 (0.588–0.621)	0.956 (0.950–0.960)	0.268 (0.222–0.319)	0.991 (0.989–0.993)	0.880 (0.861–0.898)	0.957 (0.937–0.974)
	LoRA tuning	0.797 (0.782–0.813)	0.588 (0.569–0.604)	0.955 (0.950–0.960)	0.258 (0.213–0.309)	0.992 (0.989–0.994)	0.871 (0.850–0.890)	0.950 (0.926–0.971)
VisionFM	Partial Finetuning	0.878 (0.861–0.895)	0.503 (0.483–0.522)	0.948 (0.943–0.954)	0.166 (0.129–0.211)	0.989 (0.986–0.991)	0.833 (0.810–0.854)	0.914 (0.885–0.939)
	LoRA tuning	0.803 (0.789–0.819)	0.580 (0.562–0.596)	0.952 (0.947–0.957)	0.229 (0.186–0.279)	0.990 (0.987–0.992)	0.874 (0.855–0.892)	0.952 (0.933–0.970)

Performance metrics are reported for the test dataset and include mean absolute error (MAE), for which lower values indicate better performance; and R-squared, accuracy, sensitivity for anaemia (Sens.), specificity for anaemia (Spec.), area under the receiver operating characteristic curve (AUROC), and AUROC for clinically significant anaemia, for which higher values indicate better performance. All metrics are presented with 95% confidence intervals (CIs). Clinically significant anaemia was defined as a haemoglobin concentration <11 g/dL. This test set included 51 clinically significant anaemia cases among 3458 participants (1.47%).

To further characterise the operating characteristics of the best-performing model, Table 3 reports sensitivity at predefined operating points corresponding to fixed specificity levels for any anaemia. As target specificity increased from 70% to 95%, sensitivity for any anaemia decreased from 0.930 to 0.611, reflecting the expected trade-off. In contrast, detection of clinically significant anaemia remained consistently high across these operating points, exceeding 0.95 even at higher specificity levels.

**Table 3:** Operating-point analysis at fixed specificity for any anaemia by the DINOv2 model with LoRA finetuning.

Target specificity	Hb threshold (g/dL)	Sensitivity	Sensitivity (cSA)
70	+1.436	0.930 (0.896–0.953)	1.000 (0.962–1.000)
80	+1.177	0.869 (0.828–0.902)	0.990 (0.944–0.998)
90	+0.827	0.726 (0.674–0.772)	0.959 (0.900–0.984)
95	+0.541	0.611 (0.557–0.664)	0.959 (0.900–0.984)

Operating-point analysis of the best-performing DINOv2-based model fine-tuned using LoRA at fixed specificity for any anaemia. Operating points were defined by fixing specificity with respect to any anaemia using sex-specific haemoglobin thresholds, and a single predicted haemoglobin threshold was applied across outcomes. At each operating point, sensitivity was reported for both any anaemia and clinically significant anaemia (haemoglobin < 11 g/dL). Clinically significant anaemia was defined as a haemoglobin concentration < 11 g/dL. This test set included 51 clinically significant anaemia cases among 3458 participants (1.47%).

Supplementary Fig. 2 presents the positive predictive value stratified by percentiles of predicted haemoglobin concentration. Lower predicted haemoglobin percentiles were associated with a higher prevalence of true anaemia, consistent with the interpretation of the model output as a continuous risk signal. These results remained consistent across subgroups stratified by sex, age, hypertension, diabetes mellitus, and dyslipidaemia as described in Supplementary Tables 3–9.

### Explaining the clinical rationale of the model

**Fig. 2:**
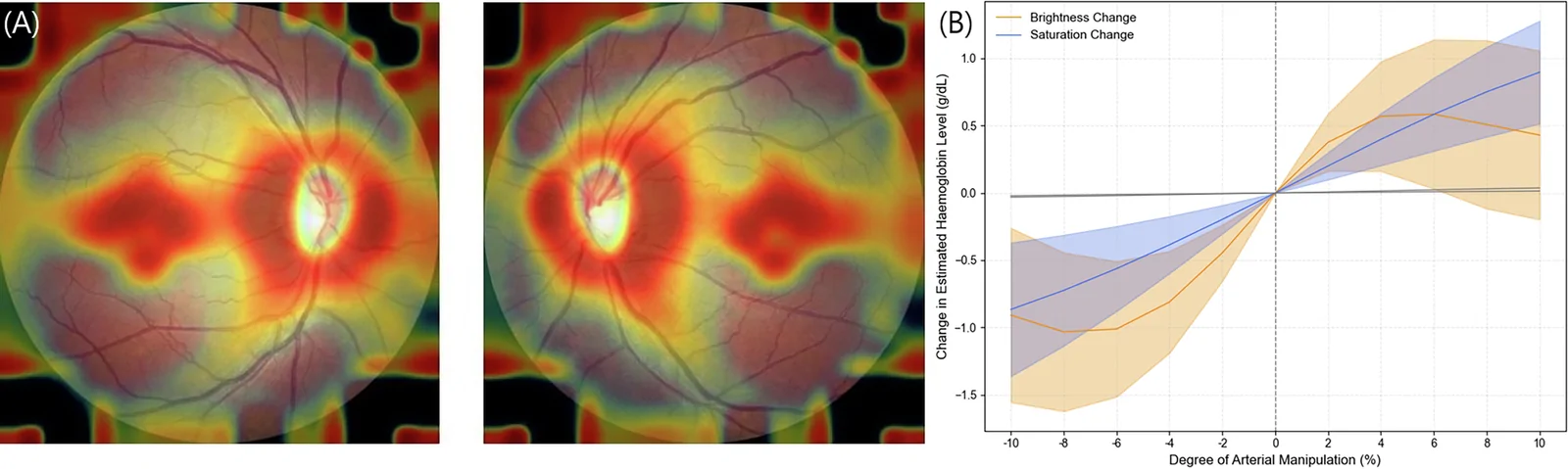
Explainability analyses for haemoglobin estimation by the DINOv2 model with LoRA fine-tuning. **A** Average Grad-CAM heatmaps of the test set showing retinal regions contributing to haemoglobin prediction. Warmer colours indicate greater importance. **B** Changes in predicted haemoglobin concentrations following arterial brightness and saturation manipulation. Shaded areas indicate standard deviations, and black lines represent equivalent modifications applied to the entire image. Representative Grad-CAM heatmap highlighting image regions contributing to haemoglobin prediction by the DINOv2 model with LoRA finetuning.

For the averaged saliency maps generated using Grad-CAM, the DINOv2 fine-tuned using LoRA model focused primarily on the macula and peripapillary regions bilaterally, as shown in Fig. 2A. As illustrated in Fig. 2B, increasing the brightness of the retinal arteries by 10% resulted in an average increase of 0.43 g/dL in the predicted haemoglobin concentration, whilst a 10% decrease in brightness resulted in a 0.91 g/dL decrease. Similarly, a 10% increase in arterial saturation yielded a 0.90 g/dL increase in predicted haemoglobin, whereas a 10% decrease caused a 0.87 g/dL decline. These results suggest that increased brightness and colour saturation of the retinal arteries are positively associated with haemoglobin concentration. In contrast, when similar brightness and saturation modifications were applied to the entire image, predicted haemoglobin levels changed minimally, indicating that the model’s response was specifically tied to arterial features rather than to arbitrary global image changes.

### Comparison with previous haemoglobin estimation methods

Supplementary Table 10 shows a summary of performance across prior models employing different methodologies. Invasive methods remained the most accurate overall, whilst deep learning-based noninvasive methods outperformed other noninvasive approaches. Our FM-based results achieved an MAE of 0.70 with a mean difference of 0.08 and 95% limits of agreement ranging from −1.84 to 1.67, demonstrating performance competitive with that of models developed from scratch.

## Discussion

Foundation models (FMs) offer key advantages in medical AI development, including scalability, efficient fine-tuning, and reduced dependence on large annotated datasets. Their strong generalisation capabilities allow rapid adaptation to new clinical tasks with minimal data and development effort [19–21]. To the best of our knowledge, this is the first study to utilise FMs to predict haemoglobin from retinal fundus images of Korean adults. Some approaches have attempted to measure haemoglobin from medical images via deep learning [13–17]. Compared with existing approaches, our FM-based method demonstrated competitive regression performance relative to prior invasive, noninvasive, and conventional deep learning-based methods, suggesting a complementary noninvasive means of haemoglobin estimation. Furthermore, FMs with adequate fine-tuning achieved performance competitive with that of models trained from scratch, supporting their potential for rapid development across diverse clinical settings. The findings suggest that FMs pretrained on natural images performed better than medical image-specific FMs in this regression task. This may be attributed to the greater availability and accessibility of high-quality training data for natural FMs. This offers insights into the transfer learning capabilities of general-purpose vision models in specialised medical applications. However, further comparative studies are necessary, as performance is influenced by model architecture, hyperparameters, and pretraining data volume. Future work should focus on large-scale and high-quality medical datasets to improve the performance of medical FMs.

For the best-performing model, DINOv2 fine-tuned with LoRA, Grad-CAM visualisations revealed consistent attention to anatomically significant regions, particularly the macula which is crucial for central vision [38, 39], and the peripapillary areas where ganglion cell axons concentrate and cotton-wool spots, indicative of retinal ischaemia, are commonly observed [40, 41]. This attention pattern suggests that the model’s predictions are based on physiologically relevant features rather than arbitrary patterns, lending clinical credibility to its decision-making process. Furthermore, the relationship between arterial characteristics and predicted haemoglobin values aligns with clinical understanding. Specifically, the model showed that higher predicted haemoglobin levels were associated with increased brightness and saturation of retinal arteries. This pattern is physiologically plausible given the muscular structure of arterial walls and the light-absorbing properties of haemoglobin [42–44]. Previous studies have reported that anaemia can affect the central retinal artery, consistent with our observations [45, 46]. This alignment between model behaviour and clinical knowledge strengthens the relevance of our approach.

From a clinical implementation perspective, our study suggests that fine-tuned FMs can estimate haemoglobin and characterise anaemia-related risk using retinal fundus images in Korean adults, consistent with previous retinal image-based haemoglobin estimation studies [47–49]. However, the model shows proportional bias with overestimation at low haemoglobin and underestimation at high levels. This may lead to falsely reassuring predictions in individuals with true anaemia. In addition, the relatively wide limits of agreement (~3.5 g/dL) warrant cautious interpretation of individual predictions. Accordingly, the model should be considered an adjunctive risk assessment tool rather than a standalone diagnostic method. Fundus photography is already routinely performed in ophthalmic practice, and this approach could be integrated into existing workflows without additional imaging or procedural costs. Haemoglobin estimation via fundus photography is noninvasive and increasingly accessible with portable or smartphone-based devices, making it suitable for remote or resource-limited settings [15, 25]. Integration into telemedicine platforms could enable risk stratification and prioritisation for clinical evaluation. When interpreted in conjunction with clinical context and laboratory confirmation, this approach may facilitate timely decision-making [3, 5].

Several limitations warrant discussion and suggest directions for future research. The primary limitation of this study is the limited generalisability of the findings. The proposed model was developed and evaluated using data from a single Korean institution acquired using a single non-mydriatic fundus camera, which poses a substantial challenge for translation to real-world clinical settings. In particular, variations in retinal pigmentation and imaging hardware may significantly affect model performance, yet these factors were not captured within the present study design. Robust external validation across diverse populations and imaging devices is therefore essential before any broader clinical applicability can be established.

Second, limitations related to internal validity and robustness should be considered. Whilst our single random split ensured reproducibility and consistent model comparison, performance estimates may be sensitive to the particular distribution of samples. Furthermore, subgroup analyses stratified by sex revealed variability in performance. Notably, some models yielded negative R^2^ values in these subgroups. As R^2^ is sensitive to the variance of the target variable, it may be disproportionately reduced in subgroups with a narrower haemoglobin distribution and thus should be interpreted with caution. Therefore, performance was interpreted using multiple metrics. Furthermore, the limited number of clinically significant anaemia (cSA) cases in our test set (*n* = 51) resulted in increased variability in the estimates of associated metrics. These findings should therefore be regarded as preliminary. Future studies incorporating cross-validation, sex-specific calibration or modelling strategies, and larger and more diverse cohorts are warranted to establish the robustness of these performance estimates.

Lastly, whilst the use of Grad-CAM and segmented region analysis provided valuable insights into the model’s decision-making process, a complete understanding of its reasoning remains unfeasible [23]. The segmented arterial region analysis was intended to examine whether vascular colour and brightness provide meaningful signals for haemoglobin estimation. However, these features alone are unlikely to be sufficient for robust prediction, and their isolated predictive contribution was not formally quantified in the present study. Rather, haemoglobin estimation is likely to depend on a combination of vascular, contextual, and global retinal features. Future studies could employ additional explainability and feature isolation methods to better disentangle the contributions of individual retinal features and to identify novel biomarkers for haemoglobin estimation.

In conclusion, this study provides preliminary evidence that fine-tuned FMs, including general-purpose vision models pretrained on natural images, can capture signals relevant to haemoglobin estimation from retinal fundus images within a controlled clinical setting. Among the evaluated approaches, the DINOv2 model fine-tuned with LoRA achieved the strongest predictive performance and demonstrated attention patterns concentrated on anatomically relevant retinal regions. These observations suggest that the model’s predictions are not dominated by overtly spurious image features. However, the interpretability analysis should be regarded as a qualitative sanity check rather than evidence of causal or mechanistic reasoning. Therefore, these findings should be interpreted in light of several limitations, including limited generalisability, variability across subgroups, and proportional bias. Accordingly, the model should be considered as an adjunctive risk assessment tool rather than a standalone diagnostic method. Overall, these findings support the feasibility of applying fine-tuned FMs to haemoglobin estimation from retinal fundus images under controlled conditions, whilst underscoring the need for rigorous external validation before broader clinical use can be considered.

Supplemental material is available at Eye’s website.

## Summary

### What was known before

- Anaemia diagnosis relies on blood sampling, which is invasive and may limit frequent or large-scale screening.
- Although foundation models (FMs) and vision transformers (ViTs) have shown promise in medical imaging, their feasibility for noninvasive haemoglobin estimation from fundus images remains unclear.

### What this study adds

- This study demonstrates that appropriately fine-tuned foundation models, particularly DINOv2 pretrained on natural images and fine-tuned using LoRA, can estimate haemoglobin noninvasively from retinal fundus images with competitive performance.
- Model interpretability analyses indicate attention to physiologically relevant regions, including the macula, peripapillary area, and retinal vasculature, supporting the model's potential role in risk assessment for anaemia.

## Supplementary information

supplementary tables and figure

## Data Availability

The datasets analysed during the current study are not publicly available due to institutional and patient privacy restrictions but are available from the corresponding author on reasonable request, subject to approval by the relevant institutional review board.
