# Supplementary material for: Foundation model-based haemoglobin concentration estimation and anaemia risk assessment from retinal fundus images
Source: Eye (Lond). 2026 Jul 22;40(15):2454–61. doi: 10.1038/s41433-026-04743-3 (PMC13675176; doi:10.1038/s41433-026-04743-3)
Supplement: Supplementary file 1 [file 41433_2026_4743_MOESM1_ESM.docx]

**Supplementary**

**Implementation details**

**Batch Size Configuration**

- LoRA fine-tuning: batch size = 8 (optimized for memory efficiency)
- Partial fine-tuning: batch size = 32 (leveraging increased throughput capability)

**Optimization Parameters**

- Optimizer: AdamW (chosen for its adaptive learning rate capabilities)
  - Weight decay coefficient: 1e-3
  - (for regularization)
  - β1 = 0.9, β2 = 0.999 (default momentum parameters)

**Learning rate schedule**

The learning rate schedule followed a two-phase approach:

1. Warm-up phase:
   - Duration: Initial 10 epochs
   - Learning rate: Linear increase from 0 to 5e-4
2. Decay Phase:
   - Duration: Remaining 90 epochs
   - Schedule: Cosine annealing decay
   - Final learning rate: 1e-6

**Training Duration and Loss Function**

- Total epochs: 100 (determined through empirical validation)
- Loss function: Mean squared error (MSE)
  - Selected for regression task optimization
  - MSE = 1/n Σ(yi - ŷi)² where yi represents the true haemoglobin value and ŷi represents the predicted value.

**Supplementary Table 1.** Characteristics of pretrained models used in this study

| Model | Modality | Num. of parameters | Pretrained domain | Dataset | Num. of pretrained image |
| --- | --- | --- | --- | --- | --- |
| DINOv2  (ViT giant) | Image | 1.14 B | Natural Image | LVD-142 M | 142 M |
| OpenCLIP  (ViT giant) | Image & Text | 1.01 B | Natural Image | LAION-2B | 2B |
| MAE  (ViT large) | image | 631 M | Natural Image | ImageNet-1k | 1.2 M |
| RetFound  (ViT large) | Image | 303 M | Retinal Fundus Image | MEH-MIDAS  Public datasets | 904K |
| VisionFM  (ViT base) | Image | 86 M | Retinal Fundus Image | Private dataset  Public datasets | 1 M |

**Supplementary Table 2.** Anaemia prevalence by cohort subgroup

|  | Train | Valid | Test |
| --- | --- | --- | --- |
| Total Subject Population | 27581 | 3472 | 3458 |
| Female, n (%) | 13496 | 1674 | 1706 |
| Mild anaemia | 707 (5.24%) | 85 (5.08%) | 96 (5.63%) |
| Moderate anaemia | 335 (2.48%) | 49 (2.93%) | 42 (2.46%) |
| Severe anaemia | 30 (0.22%) | 4 (0.24%) | 3 (0.18%) |
| Male, n (%) | 14085 | 1798 | 1752 |
| Mild anaemia | 264 (1.87%) | 26 (1.45%) | 26 (1.48%) |
| Moderate anaemia | 42 (0.3%) | 6 (0.33%) | 5 (0.29%) |
| Severe anaemia | 4 (0.03%) | 2 (0.11%) | 1 (0.06%) |
| Young age (<50), n (%) | 5901 | 738 | 745 |
| Mild anaemia | 215 (3.64%) | 28 (3.79%) | 29 (3.89%) |
| Moderate anaemia | 140 (2.37%) | 22 (2.98%) | 22 (2.95%) |
| Severe anaemia | 12 (0.2%) | 2 (0.27%) | 1 (0.13%) |
| Old age (≥ 65), n (%) | 7732 | 993 | 994 |
| Mild anaemia | 369 (4.77%) | 49 (4.93%) | 51 (5.13%) |
| Moderate anaemia | 65 (0.84%) | 11 (1.11%) | 3 (0.3%) |
| Severe anaemia | 2 (0.03%) | 1 (0.1%) | 2 (0.2%) |
| Hypertension, n (%) | 9119 | 1135 | 1178 |
| Mild anaemia | 291 (3.19%) | 27 (2.38%) | 37 (3.14%) |
| Moderate anaemia | 94 (1.03%) | 12 (1.06%) | 11 (0.93%) |
| Severe anaemia | 4 (0.04%) | 1 (0.09%) | 0 (0.0%) |
| Diabetes Mellitus, n (%) | 5071 | 654 | 622 |
| Mild anaemia | 224 (4.42%) | 29 (4.43%) | 21 (3.38%) |
| Moderate anaemia | 64 (1.26%) | 10 (1.53%) | 8 (1.29%) |
| Severe anaemia | 6 (0.12%) | 5 (0.76%) | 1 (0.16%) |
| Dyslipidemia, n (%) | 13333 | 1661 | 1631 |
| Mild anaemia | 374 (2.81%) | 37 (2.23%) | 39 (2.39%) |
| Moderate anaemia | 139 (1.04%) | 13 (0.78%) | 16 (0.98%) |
| Severe anaemia | 12 (0.09%) | 1 (0.06%) | 2 (0.12%) |

**Supplementary Figure 1.** Bland-Altman Plot and Linear Regression Analysis for Haemoglobin Concentration Estimation by DINOv2 with LoRA Fine-Tuning.
This figure visualises the agreement and correlation between the haemoglobin concentration predicted by the best-performing model, DINOv2 fine-tuned with LoRA, and the laboratory-measured haemoglobin concentration in the test dataset (n=3,458).

**Supplementary Figure 2**. Positive predictive value (PPV) stratified by percentiles of predicted haemoglobin concentration produced by the DINOv2 model with LoRA finetuning.

Percentiles were computed from the predicted haemoglobin distribution, with lower percentiles corresponding to lower predicted haemoglobin concentrations. (*) For female subjects, 1 [g/dL] was added to the predicted haemoglobin concentration to account for the sex-specific haemoglobin threshold difference defined by the World Health Organization.

**Supplementary Table 3.** Predictive performance of models for haemoglobin estimation and anaemia prediction in the female subgroup

| **Model** | **Finetuning Method** | Estimation of haemoglobin | | Prediction of anaemia | | | | |
| --- | --- | --- | --- | --- | --- | --- | --- | --- |
|  |  | MAE  (95% CI) | R squared  (95% CI) | Accuracy  (95% CI) | Sens.  (95% CI) | Spec.  (95% CI) | AUROC  (95% CI) | AUROC (cSA)  (95% CI) |
| DINOv2 | Partial Finetuning | 0.889  (0.863 - 0.913) | -0.059  (-0.135 - 0.012) | 0.919  (0.909 - 0.929) | 0.102  (0.070 - 0.145) | 0.993  (0.989 - 0.995) | 0.771  (0.740 - 0.799) | 0.881  (0.843 - 0.914) |
|  | LoRA tuning | 0.662  (0.644 - 0.680) | 0.418  (0.370 - 0.463) | 0.930  (0.921 - 0.939) | 0.379  (0.322 - 0.440) | 0.980  (0.974 - 0.984) | 0.887  (0.868 - 0.907) | 0.973  (0.957 - 0.985) |
| OpenCLIP | Partial Finetuning | 0.910  (0.885 - 0.934) | -0.100  (-0.186 - -0.023) | 0.917  (0.906 - 0.926) | 0.105  (0.074 - 0.149) | 0.989  (0.985 - 0.992) | 0.768  (0.738 - 0.798) | 0.885  (0.848 - 0.916) |
|  | LoRA tuning | 0.714  (0.696 - 0.733) | 0.324  (0.269 - 0.371) | 0.924  (0.914 - 0.933) | 0.281  (0.230 - 0.339) | 0.982  (0.976 - 0.986) | 0.851  (0.827 - 0.874) | 0.948  (0.926 - 0.967) |
| MAE | Partial Finetuning | 0.768  (0.746 - 0.789) | 0.207  (0.140 - 0.267) | 0.924  (0.914 - 0.933) | 0.246  (0.197 - 0.302) | 0.985  (0.980 - 0.989) | 0.841  (0.815 - 0.865) | 0.946  (0.924 - 0.965) |
|  | LoRA tuning | 0.696  (0.677 - 0.714) | 0.361  (0.307 - 0.411) | 0.927  (0.917 - 0.935) | 0.355  (0.299 - 0.416) | 0.978  (0.972 - 0.983) | 0.878  (0.856 - 0.900) | 0.963  (0.945 - 0.977) |
| RetFound | Partial Finetuning | 0.746  (0.727 - 0.767) | 0.266  (0.208 - 0.319) | 0.930  (0.921 - 0.938) | 0.293  (0.241 - 0.351) | 0.987  (0.982 - 0.991) | 0.848  (0.822 - 0.873) | 0.950  (0.927 - 0.967) |
|  | LoRA tuning | 0.739  (0.720 - 0.760) | 0.265  (0.203 - 0.321) | 0.928  (0.919 - 0.937) | 0.281  (0.230 - 0.339) | 0.986  (0.981 - 0.990) | 0.850  (0.822 - 0.876) | 0.935  (0.899 - 0.963) |
| VisionFM | Partial Finetuning | 0.838  (0.815 - 0.862) | 0.061  (-0.007 - 0.125) | 0.916  (0.906 - 0.925) | 0.180  (0.138 - 0.231) | 0.982  (0.977 - 0.986) | 0.799  (0.770 - 0.829) | 0.888  (0.850 - 0.922) |
|  | LoRA tuning | 0.762  (0.740 - 0.784) | 0.212  (0.153 - 0.267) | 0.923  (0.913 - 0.932) | 0.242  (0.194 - 0.298) | 0.984  (0.979 - 0.988) | 0.840  (0.816 - 0.863) | 0.930  (0.898 - 0.956) |

Performance metrics are reported for the female subgroup and include mean absolute error (MAE), for which lower values indicate better performance; and R-squared, accuracy, sensitivity for anaemia (Sens.), specificity for anaemia (Spec.), area under the receiver operating characteristic curve (AUROC), and AUROC for clinically significant anaemia (cSA), for which higher values indicate better performance. All metrics are presented with 95% confidence intervals (CIs). Clinically significant anaemia was defined as a haemoglobin concentration < 11 g/dL. This subgroup test set included 45 clinically significant anaemia cases among 1706 participants (2.64%).

**Supplementary Table 4.** Predictive performance of models for haemoglobin estimation and anaemia prediction in the male subgroup

| **Model** | **Finetuning Method** | Estimation of haemoglobin | | Prediction of anaemia | | | | |
| --- | --- | --- | --- | --- | --- | --- | --- | --- |
|  |  | MAE  (95% CI) | R squared  (95% CI) | Accuracy  (95% CI) | Sens.  (95% CI) | Spec.  (95% CI) | AUROC  (95% CI) | AUROC (cSA)  (95% CI) |
| DINOv2 | Partial Finetuning | 0.895  (0.871 - 0.919) | 0.030  (-0.028 - 0.084) | 0.979  (0.973 - 0.983) | 0.034  (0.010 - 0.117) | 0.996  (0.993 - 0.998) | 0.816  (0.755 - 0.869) | 0.903  (0.792 - 0.972) |
|  | LoRA tuning | 0.742  (0.722 - 0.761) | 0.344  (0.289 - 0.394) | 0.983  (0.978 - 0.987) | 0.293  (0.192 - 0.420) | 0.996  (0.993 - 0.998) | 0.931  (0.899 - 0.958) | 0.998  (0.996 - 0.999) |
| OpenCLIP | Partial Finetuning | 0.906  (0.881 - 0.930) | 0.020  (-0.048 - 0.082) | 0.984  (0.979 - 0.987) | 0.172  (0.096 - 0.289) | 0.998  (0.996 - 0.999) | 0.826  (0.771 - 0.872) | 0.913  (0.837 - 0.976) |
|  | LoRA tuning | 0.789  (0.768 - 0.810) | 0.264  (0.208 - 0.317) | 0.983  (0.978 - 0.987) | 0.241  (0.150 - 0.365) | 0.996  (0.993 - 0.998) | 0.911  (0.879 - 0.938) | 0.977  (0.954 - 0.997) |
| MAE | Partial Finetuning | 0.821  (0.799 - 0.842) | 0.195  (0.137 - 0.248) | 0.982  (0.977 - 0.986) | 0.155  (0.084 - 0.269) | 0.997  (0.995 - 0.999) | 0.885  (0.842 - 0.920) | 0.966  (0.920 - 0.997) |
|  | LoRA tuning | 0.782  (0.761 - 0.802) | 0.277  (0.221 - 0.330) | 0.982  (0.977 - 0.986) | 0.259  (0.163 - 0.384) | 0.996  (0.993 - 0.997) | 0.912  (0.879 - 0.939) | 0.996  (0.989 - 1.000) |
| RetFound | Partial Finetuning | 0.826  (0.802 - 0.846) | 0.198  (0.139 - 0.258) | 0.980  (0.975 - 0.984) | 0.155  (0.084 - 0.269) | 0.995  (0.992 - 0.997) | 0.887  (0.845 - 0.923) | 0.970  (0.938 - 0.994) |
|  | LoRA tuning | 0.853  (0.829 - 0.875) | 0.134  (0.071 - 0.195) | 0.981  (0.976 - 0.986) | 0.155  (0.084 - 0.269) | 0.996  (0.993 - 0.998) | 0.860  (0.815 - 0.900) | 0.963  (0.920 - 0.995) |
| VisionFM | Partial Finetuning | 0.916  (0.890 - 0.940) | -0.000  (-0.073 - 0.059) | 0.979  (0.974 - 0.984) | 0.103  (0.048 - 0.208) | 0.995  (0.992 - 0.997) | 0.820  (0.768 - 0.865) | 0.897  (0.805 - 0.973) |
|  | LoRA tuning | 0.843  (0.819 - 0.865) | 0.152  (0.090 - 0.208) | 0.980  (0.974 - 0.984) | 0.172  (0.096 - 0.289) | 0.995  (0.991 - 0.997) | 0.869  (0.816 - 0.911) | 0.981  (0.956 - 0.996) |

Performance metrics are reported for the male subgroup and include mean absolute error (MAE), for which lower values indicate better performance; and R-squared, accuracy, sensitivity for anaemia (Sens.), specificity for anaemia (Spec.), area under the receiver operating characteristic curve (AUROC), and AUROC for clinically significant anaemia (cSA), for which higher values indicate better performance. All metrics are presented with 95% confidence intervals (CIs). Clinically significant anaemia was defined as a haemoglobin concentration < 11 g/dL. This subgroup test set included 6 clinically significant anaemia cases among 1752 participants (0.34%).

| **Model** | **Finetuning Method** | Estimation of haemoglobin | | Prediction of anaemia | | | | |
| --- | --- | --- | --- | --- | --- | --- | --- | --- |
|  |  | MAE  (95% CI) | R squared  (95% CI) | Accuracy  (95% CI) | Sens.  (95% CI) | Spec.  (95% CI) | AUROC  (95% CI) | AUROC (cSA)  (95% CI) |
| DINOv2 | Partial Finetuning | 0.921  (0.886 - 0.960) | 0.579  (0.544 - 0.609) | 0.937  (0.923 - 0.948) | 0.190  (0.125 - 0.278) | 0.993  (0.986 - 0.996) | 0.868  (0.833 - 0.901) | 0.945  (0.923 - 0.966) |
|  | LoRA tuning | 0.723  (0.692 - 0.751) | 0.743  (0.716 - 0.766) | 0.951  (0.938 - 0.961) | 0.460  (0.366 - 0.557) | 0.987  (0.980 - 0.992) | 0.936  (0.914 - 0.954) | 0.980  (0.961 - 0.993) |
| OpenCLIP | Partial Finetuning | 0.946  (0.910 - 0.986) | 0.555  (0.517 - 0.588) | 0.931  (0.916 - 0.943) | 0.170  (0.109 - 0.255) | 0.987  (0.980 - 0.992) | 0.833  (0.787 - 0.871) | 0.949  (0.928 - 0.967) |
|  | LoRA tuning | 0.783  (0.753 - 0.816) | 0.700  (0.672 - 0.724) | 0.940  (0.926 - 0.951) | 0.370  (0.282 - 0.468) | 0.982  (0.974 - 0.988) | 0.902  (0.873 - 0.928) | 0.968  (0.951 - 0.982) |
| MAE | Partial Finetuning | 0.812  (0.780 - 0.845) | 0.667  (0.635 - 0.696) | 0.938  (0.925 - 0.950) | 0.310  (0.228 - 0.406) | 0.985  (0.977 - 0.990) | 0.898  (0.866 - 0.926) | 0.973  (0.956 - 0.985) |
|  | LoRA tuning | 0.753  (0.724 - 0.783) | 0.720  (0.691 - 0.744) | 0.940  (0.926 - 0.951) | 0.360  (0.273 - 0.458) | 0.983  (0.974 - 0.989) | 0.920  (0.893 - 0.944) | 0.980  (0.969 - 0.988) |
| RetFound | Partial Finetuning | 0.823  (0.790 - 0.857) | 0.671  (0.638 - 0.700) | 0.943  (0.930 - 0.954) | 0.410  (0.319 - 0.508) | 0.983  (0.974 - 0.989) | 0.903  (0.870 - 0.933) | 0.972  (0.957 - 0.984) |
|  | LoRA tuning | 0.839  (0.805 - 0.875) | 0.651  (0.616 - 0.683) | 0.941  (0.928 - 0.952) | 0.380  (0.291 - 0.478) | 0.983  (0.974 - 0.989) | 0.895  (0.863 - 0.926) | 0.971  (0.955 - 0.985) |
| VisionFM | Partial Finetuning | 0.902  (0.869 - 0.941) | 0.599  (0.566 - 0.630) | 0.931  (0.916 - 0.943) | 0.230  (0.158 - 0.322) | 0.983  (0.974 - 0.989) | 0.885  (0.858 - 0.912) | 0.939  (0.913 - 0.963) |
|  | LoRA tuning | 0.824  (0.792 - 0.861) | 0.662  (0.628 - 0.688) | 0.938  (0.924 - 0.949) | 0.350  (0.264 - 0.447) | 0.981  (0.973 - 0.987) | 0.919  (0.897 - 0.939) | 0.965  (0.946 - 0.980) |

**Supplementary Table 5.** Predictive performance of models for haemoglobin estimation and anaemia prediction in the young age(< 50) subgroup

Performance metrics are reported for the young age subgroup and include mean absolute error (MAE), for which lower values indicate better performance; and R-squared, accuracy, sensitivity for anaemia (Sens.), specificity for anaemia (Spec.), area under the receiver operating characteristic curve (AUROC), and AUROC for clinically significant anaemia (cSA), for which higher values indicate better performance. All metrics are presented with 95% confidence intervals (CIs). Clinically significant anaemia was defined as a haemoglobin concentration < 11 g/dL This subgroup test set included 23 clinically significant anaemia cases among 745 participants (3.09%).

**Supplementary Table 6.** Predictive performance of models for haemoglobin estimation and anaemia prediction in the old age(≥ 65) subgroup

| **Model** | **Finetuning Method** | Estimation of haemoglobin | | Prediction of anaemia | | | | |
| --- | --- | --- | --- | --- | --- | --- | --- | --- |
|  |  | MAE  (95% CI) | R squared  (95% CI) | Accuracy  (95% CI) | Sens.  (95% CI) | Spec.  (95% CI) | AUROC  (95% CI) | AUROC (cSA)  (95% CI) |
| DINOv2 | Partial Finetuning | 0.872  (0.837 - 0.904) | 0.390  (0.349 - 0.429) | 0.938  (0.925 - 0.949) | 0.021  (0.006 - 0.074) | 0.992  (0.987 - 0.996) | 0.765  (0.717 - 0.810) | 0.854  (0.727 - 0.963) |
|  | LoRA tuning | 0.704  (0.677 - 0.732) | 0.592  (0.548 - 0.627) | 0.940  (0.928 - 0.951) | 0.202  (0.133 - 0.294) | 0.984  (0.977 - 0.989) | 0.875  (0.842 - 0.904) | 0.971  (0.938 - 0.998) |
| OpenCLIP | Partial Finetuning | 0.889  (0.854 - 0.921) | 0.373  (0.326 - 0.415) | 0.947  (0.935 - 0.957) | 0.117  (0.067 - 0.198) | 0.996  (0.992 - 0.998) | 0.764  (0.715 - 0.814) | 0.841  (0.696 - 0.991) |
|  | LoRA tuning | 0.754  (0.726 - 0.783) | 0.546  (0.502 - 0.581) | 0.942  (0.930 - 0.952) | 0.138  (0.083 - 0.222) | 0.990  (0.984 - 0.994) | 0.850  (0.814 - 0.884) | 0.890  (0.775 - 0.995) |
| MAE | Partial Finetuning | 0.794  (0.766 - 0.824) | 0.497  (0.453 - 0.535) | 0.946  (0.934 - 0.956) | 0.128  (0.075 - 0.210) | 0.994  (0.989 - 0.997) | 0.823  (0.782 - 0.862) | 0.887  (0.769 - 0.991) |
|  | LoRA tuning | 0.743  (0.714 - 0.770) | 0.559  (0.516 - 0.596) | 0.944  (0.932 - 0.954) | 0.223  (0.151 - 0.318) | 0.987  (0.980 - 0.991) | 0.850  (0.812 - 0.885) | 0.934  (0.852 - 0.998) |
| RetFound | Partial Finetuning | 0.779  (0.749 - 0.809) | 0.517  (0.474 - 0.555) | 0.941  (0.929 - 0.951) | 0.106  (0.059 - 0.185) | 0.991  (0.984 - 0.994) | 0.819  (0.775 - 0.859) | 0.901  (0.798 - 0.991) |
|  | LoRA tuning | 0.787  (0.757 - 0.818) | 0.501  (0.453 - 0.541) | 0.945  (0.933 - 0.955) | 0.149  (0.091 - 0.235) | 0.992  (0.987 - 0.996) | 0.811  (0.765 - 0.853) | 0.892  (0.790 - 0.992) |
| VisionFM | Partial Finetuning | 0.878  (0.842 - 0.907) | 0.390  (0.337 - 0.435) | 0.937  (0.925 - 0.948) | 0.096  (0.051 - 0.172) | 0.987  (0.981 - 0.992) | 0.761  (0.711 - 0.807) | 0.845  (0.677 - 0.992) |
|  | LoRA tuning | 0.805  (0.775 - 0.835) | 0.474  (0.430 - 0.512) | 0.942  (0.929 - 0.952) | 0.117  (0.067 - 0.198) | 0.991  (0.984 - 0.994) | 0.803  (0.758 - 0.848) | 0.891  (0.791 - 0.985) |

Performance metrics are reported for the old age subgroup and include mean absolute error (MAE), for which lower values indicate better performance; and R-squared, accuracy, sensitivity for anaemia (Sens.), specificity for anaemia (Spec.), area under the receiver operating characteristic curve (AUROC), and AUROC for clinically significant anaemia (cSA), for which higher values indicate better performance. All metrics are presented with 95% confidence intervals (CIs). Clinically significant anaemia was defined as a haemoglobin concentration < 11 g/dL. This subgroup test set included 5 clinically significant anaemia cases among 994 participants (0.50%).

**Supplementary Table 7.** Predictive performance of models for haemoglobin estimation and anaemia prediction in the hypertension subgroup

| **Model** | **Finetuning Method** | Estimation of haemoglobin | | Prediction of anaemia | | | | |
| --- | --- | --- | --- | --- | --- | --- | --- | --- |
|  |  | MAE  (95% CI) | R squared  (95% CI) | Accuracy  (95% CI) | Sens.  (95% CI) | Spec.  (95% CI) | AUROC  (95% CI) | AUROC (cSA)  (95% CI) |
| DINOv2 | Partial Finetuning | 0.887  (0.857 - 0.917) | 0.440  (0.406 - 0.472) | 0.958  (0.948 - 0.966) | 0.026  (0.007 - 0.089) | 0.994  (0.989 - 0.996) | 0.793  (0.742 - 0.841) | 0.924  (0.866 - 0.973) |
|  | LoRA tuning | 0.723  (0.698 - 0.748) | 0.624  (0.595 - 0.653) | 0.965  (0.956 - 0.972) | 0.321  (0.227 - 0.430) | 0.990  (0.984 - 0.993) | 0.893  (0.855 - 0.927) | 0.979  (0.964 - 0.991) |
| OpenCLIP | Partial Finetuning | 0.911  (0.880 - 0.942) | 0.416  (0.381 - 0.450) | 0.962  (0.953 - 0.969) | 0.090  (0.044 - 0.174) | 0.995  (0.991 - 0.997) | 0.794  (0.743 - 0.842) | 0.900  (0.823 - 0.958) |
|  | LoRA tuning | 0.774  (0.749 - 0.802) | 0.575  (0.547 - 0.603) | 0.963  (0.954 - 0.970) | 0.179  (0.110 - 0.279) | 0.993  (0.988 - 0.996) | 0.882  (0.846 - 0.916) | 0.931  (0.870 - 0.976) |
| MAE | Partial Finetuning | 0.813  (0.787 - 0.842) | 0.526  (0.492 - 0.559) | 0.961  (0.952 - 0.969) | 0.167  (0.100 - 0.265) | 0.992  (0.987 - 0.995) | 0.842  (0.799 - 0.883) | 0.933  (0.873 - 0.975) |
|  | LoRA tuning | 0.759  (0.731 - 0.785) | 0.590  (0.559 - 0.620) | 0.964  (0.955 - 0.971) | 0.321  (0.227 - 0.430) | 0.988  (0.982 - 0.992) | 0.885  (0.850 - 0.917) | 0.970  (0.935 - 0.994) |
| RetFound | Partial Finetuning | 0.804  (0.779 - 0.833) | 0.545  (0.513 - 0.576) | 0.960  (0.951 - 0.968) | 0.179  (0.110 - 0.279) | 0.990  (0.985 - 0.994) | 0.866  (0.827 - 0.900) | 0.921  (0.857 - 0.971) |
|  | LoRA tuning | 0.828  (0.799 - 0.856) | 0.507  (0.473 - 0.540) | 0.961  (0.952 - 0.969) | 0.167  (0.100 - 0.265) | 0.991  (0.986 - 0.994) | 0.834  (0.790 - 0.876) | 0.910  (0.842 - 0.968) |
| VisionFM | Partial Finetuning | 0.886  (0.857 - 0.915) | 0.450  (0.414 - 0.484) | 0.957  (0.948 - 0.965) | 0.154  (0.090 - 0.250) | 0.988  (0.982 - 0.992) | 0.820  (0.776 - 0.862) | 0.904  (0.830 - 0.967) |
|  | LoRA tuning | 0.821  (0.794 - 0.849) | 0.511  (0.476 - 0.542) | 0.957  (0.947 - 0.965) | 0.128  (0.071 - 0.220) | 0.989  (0.983 - 0.992) | 0.847  (0.801 - 0.887) | 0.926  (0.878 - 0.966) |

Performance metrics are reported for the hypertension subgroup and include mean absolute error (MAE), for which lower values indicate better performance; and R-squared, accuracy, sensitivity for anaemia (Sens.), specificity for anaemia (Spec.), area under the receiver operating characteristic curve (AUROC), and AUROC for clinically significant anaemia (cSA), for which higher values indicate better performance. All metrics are presented with 95% confidence intervals (CIs). Clinically significant anaemia was defined as a haemoglobin concentration < 11 g/dL. This subgroup test set included 11 clinically significant anaemia cases among 1178 participants (0.93%).

**Supplementary Table 8.** Predictive performance of models for haemoglobin estimation and anaemia prediction in the diabetes mellitus subgroup

| **Model** | **Finetuning Method** | Estimation of haemoglobin | | Prediction of anaemia | | | | |
| --- | --- | --- | --- | --- | --- | --- | --- | --- |
|  |  | MAE  (95% CI) | R squared  (95% CI) | Accuracy  (95% CI) | Sens.  (95% CI) | Spec.  (95% CI) | AUROC  (95% CI) | AUROC (cSA)  (95% CI) |
| DINOv2 | Partial Finetuning | 0.895  (0.850 - 0.939) | 0.491  (0.445 - 0.531) | 0.953  (0.939 - 0.964) | 0.111  (0.052 - 0.222) | 0.997  (0.991 - 0.999) | 0.819  (0.759 - 0.874) | 0.934  (0.870 - 0.986) |
|  | LoRA tuning | 0.714  (0.680 - 0.750) | 0.676  (0.635 - 0.713) | 0.962  (0.949 - 0.972) | 0.500  (0.371 - 0.629) | 0.986  (0.977 - 0.992) | 0.920  (0.879 - 0.953) | 0.989  (0.974 - 0.999) |
| OpenCLIP | Partial Finetuning | 0.920  (0.876 - 0.962) | 0.470  (0.420 - 0.517) | 0.957  (0.943 - 0.967) | 0.167  (0.090 - 0.287) | 0.998  (0.993 - 0.999) | 0.808  (0.749 - 0.863) | 0.909  (0.831 - 0.975) |
|  | LoRA tuning | 0.749  (0.716 - 0.785) | 0.643  (0.606 - 0.679) | 0.960  (0.947 - 0.971) | 0.370  (0.254 - 0.504) | 0.991  (0.984 - 0.995) | 0.899  (0.858 - 0.937) | 0.957  (0.901 - 0.996) |
| MAE | Partial Finetuning | 0.802  (0.769 - 0.838) | 0.603  (0.561 - 0.643) | 0.957  (0.943 - 0.967) | 0.278  (0.176 - 0.409) | 0.992  (0.985 - 0.996) | 0.881  (0.835 - 0.925) | 0.960  (0.909 - 0.993) |
|  | LoRA tuning | 0.732  (0.699 - 0.767) | 0.658  (0.618 - 0.695) | 0.965  (0.952 - 0.974) | 0.500  (0.371 - 0.629) | 0.989  (0.981 - 0.994) | 0.910  (0.867 - 0.947) | 0.980  (0.945 - 0.998) |
| RetFound | Partial Finetuning | 0.797  (0.763 - 0.837) | 0.599  (0.554 - 0.640) | 0.961  (0.948 - 0.971) | 0.370  (0.254 - 0.504) | 0.992  (0.985 - 0.996) | 0.882  (0.829 - 0.928) | 0.969  (0.928 - 0.994) |
|  | LoRA tuning | 0.803  (0.765 - 0.841) | 0.593  (0.548 - 0.635) | 0.963  (0.950 - 0.973) | 0.333  (0.222 - 0.466) | 0.996  (0.990 - 0.998) | 0.877  (0.817 - 0.927) | 0.978  (0.954 - 0.996) |
| VisionFM | Partial Finetuning | 0.872  (0.828 - 0.914) | 0.514  (0.462 - 0.564) | 0.953  (0.939 - 0.964) | 0.259  (0.161 - 0.389) | 0.989  (0.981 - 0.994) | 0.850  (0.794 - 0.900) | 0.918  (0.834 - 0.978) |
|  | LoRA tuning | 0.796  (0.757 - 0.836) | 0.585  (0.544 - 0.625) | 0.957  (0.943 - 0.967) | 0.333  (0.222 - 0.466) | 0.989  (0.981 - 0.994) | 0.881  (0.826 - 0.929) | 0.972  (0.943 - 0.992) |

Performance metrics are reported for the diabetes mellitus subgroup and include mean absolute error (MAE), for which lower values indicate better performance; and R-squared, accuracy, sensitivity for anaemia (Sens.), specificity for anaemia (Spec.), area under the receiver operating characteristic curve (AUROC), and AUROC for clinically significant anaemia (cSA), for which higher values indicate better performance. All metrics are presented with 95% confidence intervals (CIs). Clinically significant anaemia was defined as a haemoglobin concentration < 11 g/dL. This subgroup test set included 9 clinically significant anaemia cases among 622 participants (1.45%).

**Supplementary Table 9.** Predictive performance of models for haemoglobin estimation and anaemia prediction in the dyslipidemia subgroup

| **Model** | **Finetuning Method** | Estimation of haemoglobin | | Prediction of anaemia | | | | |
| --- | --- | --- | --- | --- | --- | --- | --- | --- |
|  |  | MAE  (95% CI) | R squared  (95% CI) | Accuracy  (95% CI) | Sens.  (95% CI) | Spec.  (95% CI) | AUROC  (95% CI) | AUROC (cSA)  (95% CI) |
| DINOv2 | Partial Finetuning | 0.882  (0.857 - 0.907) | 0.485  (0.458 - 0.510) | 0.964  (0.957 - 0.971) | 0.129  (0.077 - 0.208) | 0.993  (0.990 - 0.996) | 0.843  (0.806 - 0.874) | 0.923  (0.884 - 0.959) |
|  | LoRA tuning | 0.703  (0.682 - 0.722) | 0.679  (0.656 - 0.700) | 0.968  (0.961 - 0.974) | 0.396  (0.306 - 0.494) | 0.988  (0.983 - 0.991) | 0.931  (0.908 - 0.950) | 0.987  (0.978 - 0.995) |
| OpenCLIP | Partial Finetuning | 0.898  (0.874 - 0.924) | 0.472  (0.444 - 0.496) | 0.966  (0.959 - 0.972) | 0.139  (0.084 - 0.219) | 0.995  (0.992 - 0.997) | 0.831  (0.793 - 0.867) | 0.915  (0.870 - 0.954) |
|  | LoRA tuning | 0.745  (0.723 - 0.766) | 0.633  (0.607 - 0.655) | 0.967  (0.960 - 0.973) | 0.307  (0.225 - 0.403) | 0.990  (0.985 - 0.993) | 0.903  (0.875 - 0.928) | 0.958  (0.926 - 0.987) |
| MAE | Partial Finetuning | 0.779  (0.755 - 0.802) | 0.597  (0.570 - 0.622) | 0.968  (0.961 - 0.974) | 0.287  (0.208 - 0.382) | 0.992  (0.988 - 0.994) | 0.893  (0.861 - 0.921) | 0.950  (0.916 - 0.978) |
|  | LoRA tuning | 0.732  (0.711 - 0.752) | 0.649  (0.626 - 0.671) | 0.967  (0.960 - 0.973) | 0.366  (0.279 - 0.464) | 0.988  (0.983 - 0.991) | 0.915  (0.887 - 0.937) | 0.975  (0.954 - 0.992) |
| RetFound | Partial Finetuning | 0.784  (0.762 - 0.806) | 0.595  (0.569 - 0.619) | 0.967  (0.960 - 0.973) | 0.277  (0.199 - 0.371) | 0.991  (0.987 - 0.994) | 0.888  (0.859 - 0.914) | 0.951  (0.912 - 0.981) |
|  | LoRA tuning | 0.805  (0.782 - 0.828) | 0.571  (0.541 - 0.597) | 0.967  (0.960 - 0.973) | 0.257  (0.182 - 0.350) | 0.991  (0.987 - 0.994) | 0.882  (0.850 - 0.910) | 0.932  (0.884 - 0.971) |
| VisionFM | Partial Finetuning | 0.876  (0.850 - 0.900) | 0.494  (0.464 - 0.523) | 0.962  (0.955 - 0.968) | 0.188  (0.124 - 0.275) | 0.989  (0.985 - 0.992) | 0.852  (0.817 - 0.885) | 0.901  (0.853 - 0.947) |
|  | LoRA tuning | 0.803  (0.780 - 0.827) | 0.574  (0.546 - 0.597) | 0.966  (0.959 - 0.972) | 0.297  (0.217 - 0.392) | 0.989  (0.985 - 0.992) | 0.895  (0.864 - 0.925) | 0.958  (0.926 - 0.982) |

Performance metrics are reported for the dyslipidemia subgroup and include mean absolute error (MAE), for which lower values indicate better performance; and R-squared, accuracy, sensitivity for anaemia (Sens.), specificity for anaemia (Spec.), area under the receiver operating characteristic curve (AUROC), and AUROC for clinically significant anaemia (cSA), for which higher values indicate better performance. All metrics are presented with 95% confidence intervals (CIs). Clinically significant anaemia was defined as a haemoglobin concentration < 11 g/dL. This subgroup test set included 18 clinically significant anaemia cases among 1631 participants (1.10%).

**Supplementary Table 10.** Comparison of model performance between the foundation model and conventional methods for estimating haemoglobin

|  | MAE | Mean diff. | 95% limits of agreement | |
| --- | --- | --- | --- | --- |
| Invasive point-of-care devices[47] | 0.59* | 0.08 | -1.3 | 1.4 |
| Non-invasive point-of-care devices[47] | 1.16* | -0.03 | -3.0 | 2.9 |
| Smartphone-based method[48] | 0.97 | 0.2 | -2.2 | 2.6 |
| Smartphone-based deep learning method[17] | 1.34 | 0.17* | -3.1* | 3.5* |
| Deep learning method from scratch[15,49] | 0.63 | 0.14 | -1.44 | 1.71 |
|  | 0.79 | 0.02* | -1.92 | 1.96 |
| FM with fine-tuning | 0.70 | 0.08 | -1.84 | 1.67 |

Metrices are based on evaluation using the test dataset. Asterisk(*) indicates values were estimated from published metrics. FM = foundation model; MAE = Mean absolute error; Mean diff. = Mean difference
